# Continuous Biosensing to Monitor Acute Systemic Inflammation, a Diagnostic Need for Therapeutic Guidance

**DOI:** 10.1021/acssensors.4c02569

**Published:** 2024-12-18

**Authors:** Guilherme Gouveia, Abtin Saateh, Anna Swietlikowska, Claudia Scarpellini, Emily Tsang, Hatice Altug, Maarten Merkx, Annelies Dillen, Karen Leirs, Dragana Spasic, Jeroen Lammertyn, Kurt V. Gothelf, Estelle Bonedeau, Nicola Porzberg, Ruben L. Smeets, Hans J. P. M. Koenen, Menno W. J. Prins, Marien I. de Jonge

**Affiliations:** † Department of Laboratory Medicine, Laboratory of Medical Immunology, Radboud Community for Infectious Diseases, Radboud University Medical Center, Nijmegen 6500 HB, The Netherlands; ‡ Institute of Bioengineering, École Polytechnique Fédérale de Lausanne (EPFL), Lausanne 1015, Switzerland; ∇ Laboratory of Chemical Biology, Department of Biomedical Engineering, Eindhoven University of Technology, Eindhoven 5600MB, The Netherlands; ∥ Institute for Complex Molecular Systems (ICMS), Eindhoven University of Technology, Eindhoven 5600MB, The Netherlands; ⊥ Department of Biosystems - Biosensors Group, KU Leuven, Willem de Croylaan 42, 3001 Leuven, Belgium; # Department of Chemistry and Interdisciplinary Nanoscience Center (iNANO), Aarhus University, Aarhus 8000 C, Denmark; ○ Department of Chemical Biology, Max Planck Institute for Medical Research, Jahnstrasse 29, 69120 Heidelberg, Germany; ◆ Department of Laboratory Medicine, Radboudumc Laboratory for Diagnostics, Radboud University Medical Center, Nijmegen 6500 HB, The Netherlands; ◪ Department of Biomedical Engineering, Eindhoven University of Technology, Eindhoven 5600MB, The Netherlands; ★ Department of Applied Physics, Eindhoven University of Technology, Eindhoven 5600MB, The Netherlands; ◭ Helia Biomonitoring, De Lismortel 31, 5612 AR Eindhoven, The Netherlands

**Keywords:** SIRS, CARS, sepsis, biomarkers, nanoswitches, biosensor, healthcare

## Abstract

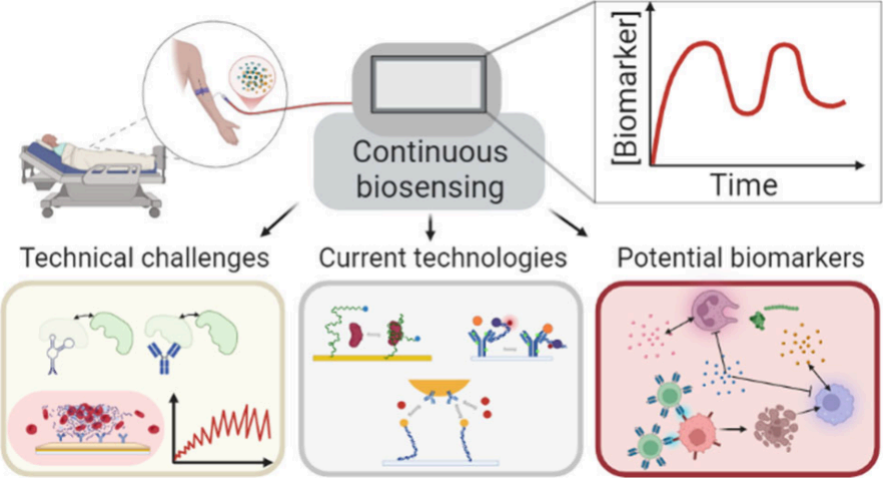

Continuous monitoring of acute inflammation can become a very important next step for guiding therapeutic interventions in severely ill patients. This Perspective discusses the current medical need for patients with acute inflammatory diseases and the potential of continuous biosensing technologies. First, we discuss biomarkers that could help to monitor the state of a patient with acute systemic inflammation based on theoretical studies and empirical data. Then, based on the state of the art, we describe sensing strategies that could be applied for the continuous monitoring of acute inflammatory biomarkers, followed by challenges that must be overcome. Nanoswitch-based continuous biosensors enable suitable measurement frequencies but still lack sensitivity, while regeneration risks lower sensor reliability. Developments are still needed in bioreceptors and molecular architectures, regeneration techniques, combined with suitable sampling and sample pretreatment methods, for bringing continuous biosensing of inflammation closer to reality. Furthermore, collaborations between healthcare professionals and scientists, regulatory bodies, and biosensor engineers are needed for a successful translation of sensing technologies from the laboratory to clinical practice.

## Acute Systemic Inflammatory Responses

### Dysregulated Immune Responses in Acute Systemic Inflammation

Inflammation is an adaptive biological response resulting from an activated immune system that serves to protect the body from environmental threats.^1^ A protective inflammatory response enforces homeostasis and preserves the structural and functional integrity of tissues and organs. The activation of an inflammatory response is caused by perturbations of homeostasis, leading to signals that relate to the harm caused by infection or injury. Molecular signals derived from pathogenic microorganisms are classified as pathogen-associated molecular patterns (PAMPs). Examples of PAMPs are lipopolysaccharide (LPS) and lipoteichoic acid, which are present in the cell walls of Gram-negative and Gram-positive bacteria, respectively. Also, nonpathogenic causes can generate inflammation, for example when tissues and cells get damaged, molecules that are usually found inside cells, like nucleic acids and actin, are released and are recognized by immune cells as damage signals. These are classified as damage-associated molecular patterns (DAMPs). Both PAMPs and DAMPs are recognized by specific receptors called pattern recognition receptors, which are ubiquitously present in and on cells. When a threat is recognized, an initial inflammatory response starts, activating effector cells to clear the cause of inflammation and restore homeostasis.^2^

The immune system senses and responds through cellular migration, interaction, and communication, leading to activation and functional responses. At the same time, negative feedback mechanisms regulate the activation in order to maintain it proportional to the threat. Therefore, inflammatory reactions can be divided into pro-inflammatory processes which help to maintain and increase inflammation, and anti-inflammatory processes involved in dampening and terminating inflammation. Soluble factors such as cytokines and acute-phase proteins are central to these mechanisms.^3^ However, perturbations of homeostasis triggered by damage-causing events such as acute infection can also lead to immune system dysregulation. This results in an ineffective clearance and collateral damage, which increase the amount of PAMPs and DAMPs respectively, leading to a hyperactivation of the immune system and acute systemic inflammation.^4^ Such dysregulation occurs, for instance, in patients developing sepsis, causing 11 million deaths worldwide each year,^5,6^ but it can also be induced by adverse reactions to immunotherapies, medication, trauma related to surgical procedures, exacerbations of autoimmune diseases, or burns.

### Dynamics in Acute Systemic Inflammatory Responses

Depending on the causes and consequences of inflammation, different systemic inflammatory syndromes have been described. They can be categorized into two groups based on symptoms and clinical presentation: Systemic Inflammation Response Syndrome (SIRS) and Compensatory Anti-inflammatory Response Syndrome (CARS). SIRS represents the primary and excessive pro-inflammatory initial insult, which arises within hours to days after the insult. It includes conditions such as sepsis, septic shock, or toxic shock, which are caused by severe invasive infection.^7^ In extreme cases, disseminated intravascular coagulation can occur, characterized by general clotting of blood vessels, which subsequently leads to (multi)organ failure and potentially to the death of the patient.^8^ SIRS can also have noninfectious causes, including cancer immunotherapies, resulting in an acute systemic inflammatory response such as cytokine release syndrome (CRS) or immune effector cell-associated neurotoxicity syndrome (ICANS), with symptoms that range from hallucinations to cerebral edema and death.^7,9,10^ The increase in availability and application of cancer immunotherapies like chimeric antigen receptor (CAR) T cell therapy has dramatically improved the survival of blood cancer patients.^11^ However, due to the high prevalence of syndromes like CRS and ICANS, which have a prevalence of 20–70%, understanding their dynamics becomes increasingly important.^10^

SIRSs can be accompanied by CARS, a compensatory response. CARS represents a secondary phase of systemic inflammation, during which the immune system responsiveness is significantly suppressed or paralyzed, which can arise within days to a couple of weeks after the initial insult. Consequently, such a state can lead to the reactivation of latent infections and increase the vulnerability to secondary infections, including opportunistic or hospital-acquired (nosocomial) infections. This can, in turn, lead to more inflammation and damage. Sepsis,^12^ COVID-19^13,14^ and surgery^15,16^ have shown to lead to immunoparalysis. Together, SIRS and CARS capture the complexity and dynamics of acute systemic inflammatory diseases.^17^

**Figure 1:**
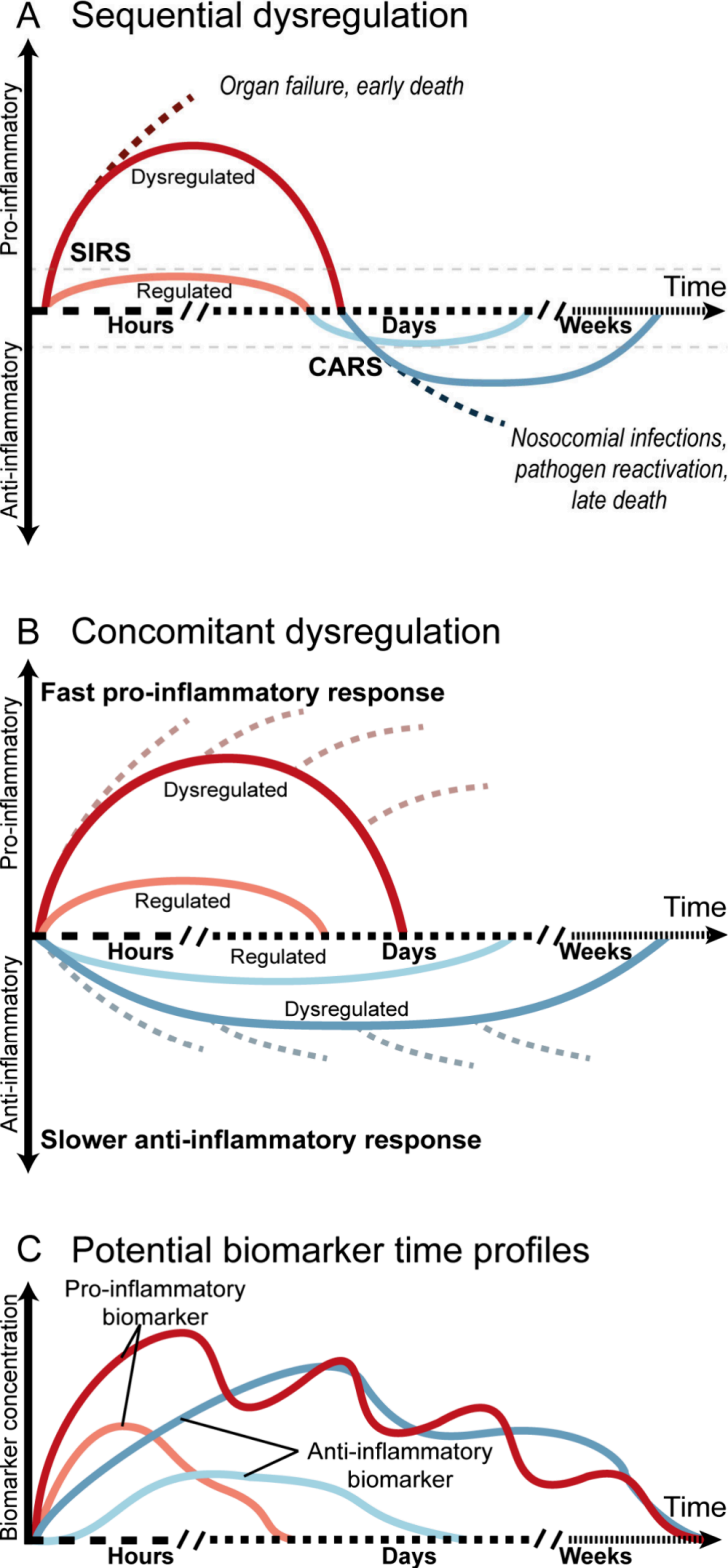
(A) Symptom-based model of acute systemic inflammation with sequential dysregulation. A first pro-inflammatory response characterized by overactivation of the immune system (SIRS) is followed by a period of immunoparalysis (CARS). The initial pro-inflammatory syndrome lasts for hours to days while the following anti-inflammatory compensation lasts from days to weeks. (B) Symptom-based model of acute systemic inflammation with concomitant dysregulation. Inflammation is balanced between both pro- and anti-inflammatory mechanisms, which act simultaneously. Due to the dysregulated nature of acute systemic inflammation, the balance can be lost within the first hours or days, due to overwhelming inflammation or excessive immunosuppression. (C) Sketch of potential time-profiles of both pro-inflammatory and anti-inflammatory biomarkers during acute systemic inflammation.

SIRS and CARS can happen sequentially, as sketched in Figure 1A or partly at the same time as pointed out in Figure 1B. While helpful in describing the trajectory and pathophysiology of patients in acute systemic inflammation, the model in Figure 1A is mainly based on observed symptoms.^18^ Current models suggest concomitant or co-occurring pro- and anti-inflammatory mechanisms (see Figure 1B) as this could give maximal efficacy in removing threats while limiting collateral damage. The lack of balance between the responses is what causes acute inflammation disorders. This is supported by changes in cellular composition and the reprogramming of their behavior, resulting in anti-inflammatory effects during the initial pro-inflammatory response.^19^ The causes of dysregulation do not solely depend on a high initial response. For example, in the case of sepsis, if the initial inflammatory response cannot effectively eliminate the infection, the sustained stimulation by PAMPs will exacerbate the immune response. Alternatively, when the anti-inflammatory mechanisms are not able to control the initial pro-inflammatory response, the collateral damage that ensues might be able to sustain it with the release of DAMPs, also leading to dysregulation. Further categorizations into “endotypes” (subtypes of a disease condition) based on different patterns, has been extensively studied in sepsis patients, based on physical symptoms, flow cytometry data, transcriptomics and/or proteomics; see a summary by van der Poll et al.^19^ Other than sepsis, endotypes in COVID-19^20^ and trauma^21^ patients have also been described, suggesting that the concept of endotypes can be generalized for other acute systemic inflammation disorders. However, these endotypes are based on symptomatic and molecular fingerprints and do not yet take into account how a patient’s condition can progress through different stages, leading to further heterogeneities in clinical presentation.^22,23^ This calls for methodologies to better quantify and analyze the time dependencies of patient conditions in case of acute systemic inflammation.

### Need for Continuous Monitoring of Acute Systemic Inflammation

The mix of pro- and anti-inflammatory mechanisms leads to overlapping characteristics of physical symptoms such as heart rate, blood pressure, respiratory rate, and body temperature, which implies that it is impossible to determine or predict the transition from a pro-inflammatory to an immunoparalysis state based solely on these symptoms. However, the symptoms are caused by underlying cellular and molecular mechanisms that can be used as biological indicators or biomarkers with fluctuating levels during different stages of acute systemic inflammation, as shown in Figure 1C. The graph sketches how levels of pro- and anti-inflammatory biomarkers could fluctuate in different phases of the disease. Biomarkers are indicative and potentially predictive of the immune status and will be discussed more thoroughly in the next section.

In acute settings, extensive analyses such as multiplex measurements of biomarkers and immune cell phenotypes are not performed, because the analysis techniques require specialized personnel and centralized laboratory facilities, which give long turnaround times, are not available 24/7, and in many hospitals are not available at all. Therefore, preventive treatments are based on generalized guidelines and lack personalization,^24,25^ not taking into account the heterogeneity of the conditions. As a result, some patients are given treatments, that were, in retrospect, not needed, unsuitable, or even harmful due to secondary effects.^26,27^ During an acute systemic-inflammatory response, a patient’s condition can change from mild to life-threatening in a matter of hours. A proactive protocol is therefore needed to prevent severe organ damage and mortality. Sensing systems, able to continuously monitor the immune-inflammatory dynamics, could give much more information than single-time point data of the immune state. This may guide physicians in applying the correct therapy at the right time, using the increase and decrease in the concentration of biomarkers for the personalization of therapies, potentially filling an important gap in present-day patient care.

### Biomarkers for Monitoring Acute Systemic Inflammation

To monitor the dynamics of acute systemic inflammation, the most suitable biomarkers are the soluble factors involved in immune signaling, like cytokines and acute phase proteins. These are secreted and, hence, are easily accessible for measurements from bodily fluids.^4,28−30^ Despite the large number of extracellular molecules involved in regulating the immune system, not all are suitable as biomarkers for predicting or monitoring the inflammatory state of a patient or for discriminating between a normally regulated and dysregulated response. An ideal biomarker must be produced early in the acute inflammatory response, be present throughout the different stages and have a short half-life, with the concentration rising and falling with progression of the disease.

The standard matrix for measuring inflammatory biomarkers is blood. Some biomarkers are present in less invasive matrices, such as interstitial fluid^64^ and saliva.^65^ However, the low correlations of protein biomarker levels between blood and saliva^66^ and the time delay between interstitial fluid and blood levels^64^ makes blood still the preferred matrix for measurements related to the patient’s inflammatory state.

There is limited data on time-dependent fluctuations of soluble protein biomarkers in the context of inflammatory diseases. One of the reasons is that high-frequency measurements are expensive and take significant time on present-day analyzers. Furthermore, the current state of technology does not easily allow to perform studies on time-dependent and patient-dependent dynamics of the immune system. Furthermore, the present-day analytical infrastructure is not suited for continuous monitoring, which we think is the most important reason for the limited amount of clinical data on biomarker dynamics. Nevertheless, valuable insights into biomarker dynamics are available from research using the human endotoxemia model.^67−69^ The model studies are based on injecting healthy human (male) volunteers with endotoxins which induce an acute immune reaction with a controlled starting point and continuous access to the subjects, allowing frequent sampling and the reconstruction of biomarker dynamics. Based on the potential to serve as proxies for a patient’s immune status, biomarkers are listed in Table 1. The listed biomarkers, cytokines or acute phase proteins, are directly involved in acute inflammation. Using data from clinical studies and the aforementioned experimental human endotoxemia model, the concentration ranges and time scale of fluctuation (how fast significant changes in concentration are observed) are indicated. It is, however, important to note that there is a larger pool of potential biomarkers (e.g., interferon γ,^70^ ferritin,^52,71,72^ soluble urokinase plasminogen activator receptor^73^). The lack of empirical data on their time-dependent fluctuations is the main reason why they are not listed in Table 1.

**Table 1:** Potential Biomarkers for the Continuous Monitoring of Acute Inflammation

biomarker	MW^a^ (kDa)	function in acute inflammation	potential clinical utility for continuous monitoring	concentration range^b^	time scale of fluctuations (h)
Interleukin 6	22.8^31^	One of the first cytokines produced upon infection or injury directly initiates the production of acute-phase proteins in the liver and indirectly propagates inflammation from a local to a systemic state.	Rapid increase suggests acute systemic inflammation, while a decrease can indicate improvement.	<0.036 to >400 pM^32−42^	0.5–1^43−48^
Tumor Necrosis Factor α	52.1^49^	One of the first cytokines produced upon infection or injury, promoting vasodilation, endothelial activation, and the recruitment of immune cells.	Persistent increase suggests worsening of inflammation. Increase after peak suggests reactivation of inflammation.	<0.059 to >58.8 pM^50^	0.5–1^43−47^
Interleukin 8	16.8^51^	Cytokine produced early after infection or injury, recruits and attracts neutrophils to the site of infection or tissue injury.	Persistent increase suggests worsening of inflammation Increase after peak suggests reactivation of inflammation.	<0.625 to >125 pM^35,36,42,52,53^	0.5–1^43,44^
Interleukin 10	18.5^54^	Cytokine produced shortly after initial response to infection or injury with an anti-inflammatory activity, regulating the immune response to preventing excessive damage.	Increase suggests the start of anti-inflammatory response. Consistently elevated levels indicate immunosuppression.	<0.054 to >54 pM^33,35,42,55,56^	0.5–1^43,44,47^
Procalcitonin	14.5^57^	Involved in the amplification of the immune response, mostly induced by PAMPs. Common inflammatory marker with a relatively long half-life (∼24 h).	Initial spike suggests bacterial infection. Gradual decrease can guide antibiotic therapy.	<3.85 to >7690 pM^33,38,56,58−61^	6–24^62^
C-reactive Protein	231.3^63^	Produced in the liver in response to IL-6 stimulation. Binds to pathogens and damaged cells to facilitate their clearance by immune cells. Most common inflammatory marker with a relatively long half-life (∼19 h).	Persistent or rising levels may indicate ineffective therapy. Gradual decrease indicates response to therapy.	<8.7 to >4350 nM^52,53,56,58^	1–24^48^

a Molecular weights refer to native, multimeric forms.

b Concentration range in both healthy and diseased.

## Continuous Biosensing as a Solution

### Requirements for Continuous Biosensing of Acute Inflammation

Biosensors that would continuously monitor the fluctuations of biomarkers during acute systemic inflammation have certain performance requirements. Time-related performance requirements for these biosensors are the monitoring frequency in relation to biomarker fluctuations, and the delay time of the sensor in relation to the medical process. Based on the Nyquist-Shannon sampling theorem,^74^ the monitoring frequency should be at least twice the highest fluctuation frequency of the biomarker in order to be able to capture relevant changes. The delay time of the biosensor is the difference between the time at which a biomarker concentration is reported by the biosensor and the time at which the patient had that biomarker concentration in their body.^75^ The delay time should be short enough not to hinder the medical process, so less than the allowed waiting time before medical action is taken. Another essential characteristic relates to the operational lifetime, i.e. the length of time during which the biosensor operates continuously without interruption or maintenance. The operational lifetime of a suitable sensor should be longer than critical disease episodes of patients. As such, we define continuous biosensors as sensors that specifically sense a biomarker with a frequency greater than twice the fluctuation time, have a delay time that is short compared to the time until medical action, and have an operational lifetime that is long with respect to typical critical disease episodes of patients. Based on the limited data available on the fluctuations of inflammatory biomarkers, we assume that the frequency of measurement should be more than once per 30 min for most biomarkers, the delay time should be less than 30 min, and the operational lifetime should be more than 12 h (Table 1). Biosensors should reliably measure concentrations above the baseline and be sensitive enough to report significant changes in concentration. Signal parameters such as the rate of change, can then be used for identification of acute systemic inflammation.

Rigorous testing protocols are needed to quantify the time-related performance of continuous biosensors. These include exposure of the biosensor to repeated increasing and decreasing step changes of biomarker concentrations, and studies of the concentration measurement precision and accuracy over long time spans.^76−78^ Finally, ELISA and bead-based immunoassays remain gold-standard methods for measuring inflammatory biomarkers. Correlation studies of biosensor data to gold standards are a key step in technology development and in the translation process toward clinical research and clinical practice.

### Continuous Biosensor Development, State-of-the-Art

**Figure 2:**
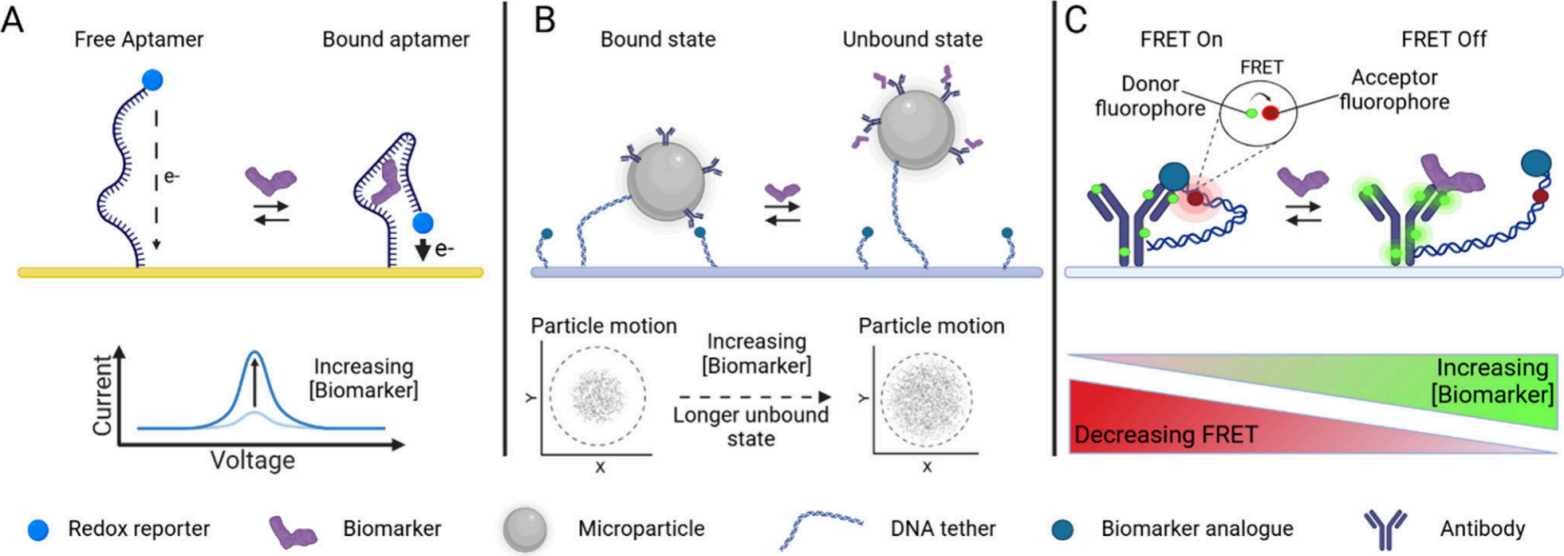
(A) Sensing principle of electrochemical aptamer-based biosensing (E-AB). The binding of a biomarker molecule to the aptamer induces a conformational change bringing the redox reporter closer to the electrode surface, increasing the measured current. Reproduced from ref (82). Copyright 2009 American Chemical Society. (B) Biosensing by particle motion (BPM), exemplified for a competitive sensor format. The particle is biofunctionalized with antibodies, and the surface is biofunctionalized with a biomarker-analogue. The particle is attached to the surface by a dsDNA tether. In the absence of biomarker molecules, the particle has a high probability to be in a bound state, due to the affinity of the antibodies to the biomarker-analogue. In the presence of biomarker, biomarker molecules bind to the antibodies, which increases the probability that a particle is in an unbound state. The state of the particle is determined by monitoring its *xy*-positions and motion properties using video microscopy. In the bound state, the motion of the particle is more restricted than when it is in the unbound state. Reproduced from ref (89). Copyright 2022 American Chemical Society. (C) Förster resonance energy transfer (FRET) nanoswitch biosensor. A biomarker analogue with acceptor fluorophore (red) is connected by a flexible DNA tether to an antibody labeled with donor fluorophores (green). In the absence of biomarker molecules, the analogue remains attached to the antibody, so the donor and acceptor fluorophores are in close proximity and allow FRET to occur. With an increase in biomarker concentration, the fluorescence emitted by the acceptor fluorophore decreases while the emission by the donor fluorophore increases, as the biomarker competes with the analogue for binding to the antibody. Reproduced with permission from reference (90). Copyright 2023 Science. Created with BioRender.com.

Biosensing is a large field of science wherein many technological approaches are being studied, with different bioreceptors, molecular constructs, detection methods, device principles and sampling methods. Biosensing systems typically consist of two parts: a part that is reused (the reader unit) and a part that is replaced (the cartridge or the sensor front-end). Traditional biosensors require a new cartridge for every new sample. In contrast, continuous sensors receive samples and measure continuously in one and the same cartridge. The dominant transduction methods used in continuous sensors to translate biomolecular interactions into measurable signals are electrochemical and optical.^79,80^ Electrochemical methods measure currents that relate to enzymatic conversion, redox currents, or changes of charge. Optical methods measure properties like refractive index, fluorescence, or scattering. In this Perspective, we do not intend to review the whole field of biosensing. Instead, we describe three examples of technologies that have been developed over the past years, making strides toward enabling continuous measurements of biomarkers at low concentrations. The three examples make use of nanoswitch principles, where molecular binding causes a switching behavior that can be electrically or optically measured, see Figure 2. Nanoswitches are an upcoming concept for continuous biosensing because such sensors can operate without consuming or producing any reagents, so they are, in principle, suited for long-term use. A redox-based nanoswitch principle for continuous biosensing was demonstrated by Plaxco et al. in 2005 and named electrochemical aptamer-based sensor (E-AB).^81^ The sensor consists of an aptamer immobilized on a gold surface and labeled with a redox reporter. Upon target binding, a conformational change occurs, inducing a change in the distance between the redox reporter and the sensor surface, see Figure 2A.^82^ This was used to measure cocaine, with real-time measurement in the high micromolar range. Over the past 20 years, the technology has steadily developed, and many research groups have been making contributions. Examples of recent developments are strategies for reducing sensor fouling in blood,^83,84^ the usage of reference zones to compensate for signal decay,^85^ the increase of the signal-to-noise ratio by using nanoporous gold electrodes^86^ and the use of xenonucleic acids^87^ to increase the lifetime of the biosensor. An example of a result is the measurement of procaine, *in vivo*, in the brains of freely moving rats.^88^

Continuous biosensing based on particle switching was presented in 2018 by Prins et al. Biosensing by particle motion (BPM) is a continuous sensing technology with hundreds to thousands of biofunctionalized particles that interact with a biofunctionalized sensing surface. The microparticles and sensor surface are functionalized with bioreceptors such as DNA or antibodies, and the motion behavior of the particles is tracked by video microscopy. Changes in particle motion are detected and the switching of the particles between unbound and bound states depends on the analyte concentration in solution, see Figure 2B for a competition-type BPM sensor. The method has single-molecule resolution because single-molecule interactions cause detectable changes in particle motion. Small molecules, such as creatinine and cortisol (in the concentration range of μM and nM, respectively), and macromolecules, such as single stranded DNA (pM concentration range), have been measured with response times ranging from minutes to hours.^100,101,105,109^ A cortisol BPM immunosensor^89^ was demonstrated that measures applied concentration changes with a time constant of 90 s^75^ and sensor stability has been studied over days.^110^

Förster resonance energy transfer (FRET) in nanoswitches has been developed for continuous biosensing by Soh et al.^90^ The nanoswitches contain fluorescent donors and acceptors, a linker molecule, and a site for specific binding of the analyte. The fluorescence signal changes upon analyte binding, see Figure 2C for a competition-type sensor. The monitoring of digoxigenin and cortisol (both nM-mM range) was shown in undiluted plasma, with a response time of approximately 5 min. The sensitivity can be tailored by tuning the affinity of the molecular competitor. To provide a platform suitable for continuous monitoring, the researchers immobilized nanoswitches on the surface of a fiber-optic sensor. Continuous FRET-based sensors have also been developed based on aptamer switches with tunable kinetic and thermodynamic properties.^113^

Although major advancements have been made in the development of continuous biosensors, it is not yet possible to continuously measure inflammatory biomarkers in blood with the necessary sensitivity and time characteristics to fill the diagnostic gap in acute systemic inflammation disorders. The main challenges that need to be overcome for this application are discussed below.

## Challenges for Continuous Biosensing in Acute Inflammation

### Improving Bioreceptors for Nanoswitches

Different possibilities exist when choosing a bioreceptor. Antibodies and their fragments,^114^ nanobodies^115^ and aptamers have been used as bioreceptors in biosensors. However, their application toward continuous biosensing requires a change in paradigm. One of the main challenges in developing continuous biosensors is the paradox of affinity requirements. High affinities are required for low limits of detection, which contrasts with the low affinities required for spontaneous release of biomarker molecules, which is necessary for fast response times. In the past, the development and screening of bioreceptors was focused on high affinity, slow dissociating antibodies and aptamers, which makes it difficult to attain reversibility.^116,117^ To obtain the required sensitivity with high specificity and low affinity, *in silico* protein^118−121^ and aptamer^121,122^ rational design or a combination of both,^112^ in combination with directed evolution^123^ can be used to complement current methods. Sensing principles, such as the examples mentioned in Table 2, can build on these improved bioreceptors to further enhance the capabilities of continuous biosensors.

**Table 2:** Properties of the Reversible Nanoswitch-Based Continuous Sensing Methods Sketched in Figure 2

biosensing concept	measured molecules	advantages	challenges	references
Electrochemical aptamer-based sensor (E-AB)	Cocaine, doxorubicin, kanamycin, gentamicin, vancomycin, tobramycin, phenylalanine, irinotecan, procaine, ampicillin, methotrexate, thrombin, ATP, neutrophil gelatinase-associated lipocalin	Demonstrated for many small molecules. E-AB sensors have been integrated on wires and microneedles. Suited for wearable sensing devices. Animal studies have been demonstrated.	Specific aptamer conformation switching properties are required. Challenges for proteins and very low concentrations.	(81−88, 91−99)
Biosensing by particle motion (BPM)	DNA, thrombin, creatinine, cortisol, glycoalkaloids, tumor necrosis factor α, lactoferrin	Particles give large optical signals. Detection method with single-molecule resolution, for measuring low concentrations (nanomolar, picomolar). Demonstrated with antibodies and aptamers as bioreceptors.	Combine low-concentration biomarkers with reversibility. Nonspecific interactions of particles and surface need to be low.	(75, 77, 89, 100−110)
FRET nanoswitch sensing	Digoxigenin, cortisol, thrombin, cAMP	Sensors have been integrated on optical fibers. Demonstrated with antibodies and aptamers as bioreceptors.	Fluorophores can be susceptible to photobleaching. FRET signals can be small.	(90, 111, 112)

### Reversibility by Sensor Regeneration

Alternatively, reversibility can be achieved by regenerating the sensor by breaking the bonds between analyte and bioreceptor molecules. Dissociation of intermolecular bonds can be done chemically, using an elution buffer, or nonchemically through methods based on acoustic fields (e.g., ultrasound waves), magnetic fields, electrical fields, electrical currents, light or temperature changes, for example.^124−127^ Regeneration methods may expand the range of suitable bioreceptors for continuous biosensing, including those with strong binding affinities. However, the regeneration will require an extra step in the sensing process that can introduce variabilities and may affect the long-term stability of the bioreceptors. Studies will aim to understand the underlying mechanisms, develop suitable sensor integrations, and characterize the regeneration effectiveness, reproducibility, and long-term stability of sensors with a variety of bioreceptors.

### Sensor Stability with Blood as the Measurement Matrix

One of the most important hurdles to overcome is the complexity of blood as the measurement matrix. Nonspecific adsorption of proteins present in blood, like albumin, globulins, lipoproteins, and fibrinogen, can create a layer on the sensing surface. As part of the coagulation process in blood, fibrinogen is converted to fibrin by thrombin, and a fibrin mesh starts to form that can trap biomolecules and even cells.^8,128^ Furthermore, protein- and nucleic acid–based bioreceptors can be degraded by proteases and nucleases present in blood. These phenomena can lead to changes in the sensor signal that are not related to the binding of the biomarker.

The sampling method should provide long-term and continuous access to blood, while allowing low volumes to be sampled. Peripherally inserted central venous catheters, are an example of an existing clinical tool that could be adapted for continuous sampling. Continuous sample pretreatment may be achieved utilizing a microdialysis probe^107^ or a filtering chamber,^129^ which reduces the complexity of the measurement matrix prior to contact with the biosensor surface. Furthermore, the sensor may be protected using antifouling layers and using degradation resistant bioreceptors in order to increase the operational lifetime.^83,84,87,130,131^

## Conclusion and Outlook

We described the background and burden of acute systemic inflammation and explained why mortality rates are still high. Current analytical technologies do not meet the clinical requirement to monitor inflammatory responses for timely and effective therapy guidance. Continuous biosensors have the potential to improve personalized therapy for patients suffering from these heterogeneous disorders for which accurate treatment requires perfect timing. However, several hurdles still need to be overcome. Sensitive and continuous measurements are challenging due to the low concentrations of the potential biomarkers. Bioreceptor development is key and can benefit from advances in screening technologies and *in silico* design. Biosensors based on nanoswitches are being developed and hold great promise for achieving high-frequency measurements of soluble biomarkers. Regeneration methodologies may also enable continuous biosensing while raising challenges on long-term use and integration. Combining continuous biosensors with current medical equipment will require significant attention and the sensors will need to be integrated with reliable sampling methods. Another major hurdle is the compatibility with blood as measurement matrix. The intrinsic complexity of blood needs to be addressed as it can be an impediment for achieving accurate long-term measurements. Continuous sample pretreatment and sensor surface modifications are some of the solutions that can help to mitigate this problem.

Ideally a continuous biosensing technology would be modular and suited for a wide variety of biomarkers. However, the different sizes of the biomarker molecules, their biochemical properties, concentration ranges and fluctuation times will require different sensor implementations, e.g. with different types of bioreceptors, coupling methods, and antifouling layers.

Thus, the biosensors should be developed with a specific biomarker application in mind, to tailor the requirements and specifications of the biosensors to the clinical need. This requires collaborations between healthcare professionals, biomedical researchers, and biosensor engineers. The availability of continuous biosensors will aid investigations into immune dynamics and will reveal characteristic time-dependencies of biomarker levels that can be used for real-time clinical feedback. The resulting information can be used to design and develop continuous sensors that can be applied to improve the monitoring and treatment of patients. This positive feedback loop of knowledge, where continuous biosensors enhance our understanding of acute systemic inflammation disorders and, in turn, support the development of applicable continuous biosensing technologies, is expected to solve important diagnostic and therapeutic challenges for patients with acute systemic inflammatory disorders.

## Vocabulary

BPM: Biosensing by Particle Motion
CAR: Chimeric antigen receptor
CARS: Compensatory Anti-inflammatory Response Syndrome
CRS: Cytokine Release Syndrome
DAMP: Damage Associated Molecular Pattern
E-AB: Electrochemical Aptamer-Based Sensor
FRET: Förster Resonance Energy Transfer
ICANS: Immune Effector Cell-associated Neurotoxicity Syndrome
LPS: Lipopolysaccharide
PAMP: Pathogen Associated Molecular Pattern
SIRS: Systemic Inflammatory Response Syndrome

## References

[ref1] Netea M. G.; Balkwill F.; Chonchol M.; Cominelli F.; Donath M. Y.; Giamarellos-Bourboulis E. J.; Golenbock D.; Gresnigt M. S.; Heneka M. T.; Hoffman H. M.; et al. A guiding map for inflammation. Nature Immunology 2017, 18 (8), 826–831. 10.1038/ni.3790. 28722720 PMC5939996

[ref2] Medzhitov R. The spectrum of inflammatory responses. Science 2021, 374 (6571), 1070–1075. 10.1126/science.abi5200. 34822279

[ref3] Sherwood E. R.; Toliver-Kinsky T. Mechanisms of the inflammatory response. Best Practice & Research Clinical Anaesthesiology 2004, 18 (3), 385–405. 10.1016/j.bpa.2003.12.002. 15212335

[ref4] Panoskaltsis N. Are all cytokine storms the same?. Cancer Immunol Immunother 2021, 70 (4), 887–892. 10.1007/s00262-020-02822-2. 33416946 PMC7791954

[ref5] Fleischmann-Struzek C.; Mellhammar L.; Rose N.; Cassini A.; Rudd K. E.; Schlattmann P.; Allegranzi B.; Reinhart K. Incidence and mortality of hospital- and ICU-treated sepsis: results from an updated and expanded systematic review and meta-analysis. Intensive Care Medicine 2020, 46 (8), 1552–1562. 10.1007/s00134-020-06151-x. 32572531 PMC7381468

[ref6] Rudd K. E.; Johnson S. C.; Agesa K. M.; Shackelford K. A.; Tsoi D.; Kievlan D. R.; Colombara D. V.; Ikuta K. S.; Kissoon N.; Finfer S.; et al. Global, regional, and national sepsis incidence and mortality, 1990–2017: analysis for the Global Burden of Disease Study. Lancet 2020, 395 (10219), 200–211. 10.1016/S0140-6736(19)32989-7. 31954465 PMC6970225

[ref7] Fajgenbaum D. C.; June C. H. Cytokine Storm. New England Journal of Medicine 2020, 383 (23), 2255–2273. 10.1056/NEJMra2026131. 33264547 PMC7727315

[ref8] Popescu N. I.; Lupu C.; Lupu F. Disseminated intravascular coagulation and its immune mechanisms. Blood 2022, 139 (13), 1973–1986. 10.1182/blood.2020007208. 34428280 PMC8972096

[ref9] Lee D. W.; Santomasso B. D.; Locke F. L.; Ghobadi A.; Turtle C. J.; Brudno J. N.; Maus M. V.; Park J. H.; Mead E.; Pavletic S.; et al. ASTCT Consensus Grading for Cytokine Release Syndrome and Neurologic Toxicity Associated with Immune Effector Cells. Biol. Blood Marrow Transplant 2019, 25 (4), 625–638. 10.1016/j.bbmt.2018.12.758. 30592986 PMC12180426

[ref10] Sterner R. C.; Sterner R. M. Immune effector cell associated neurotoxicity syndrome in chimeric antigen receptor-T cell therapy. Front Immunol 2022, 13, 879608 10.3389/fimmu.2022.879608. 36081506 PMC9445841

[ref11] Cappell K. M.; Kochenderfer J. N. Long-term outcomes following CAR T cell therapy: what we know so far. Nature Reviews Clinical Oncology 2023, 20 (6), 359–371. 10.1038/s41571-023-00754-1. PMC10100620 37055515

[ref12] Torres L. K.; Pickkers P.; van der Poll T. Sepsis-Induced Immunosuppression. Annu. Rev. Physiol. 2022, 84, 157–181. 10.1146/annurev-physiol-061121-040214. 34705481

[ref13] Zhou Y.; Liao X.; Song X.; He M.; Xiao F.; Jin X.; Xie X.; Zhang Z.; Wang B.; Zhou C.; et al. Severe Adaptive Immune Suppression May Be Why Patients With Severe COVID-19 Cannot Be Discharged From the ICU Even After Negative Viral Tests. Frontiers in Immunology 2021, 12, 12 10.3389/fimmu.2021.755579. PMC8640185 34867988

[ref14] Liu Y.; Li Y.; Xu D.; Zhang J.; Peng Z. Severe COVID-19: Immunosuppression or Hyperinflammation?. Shock 2021, 56 (2), 188–199. 10.1097/SHK.0000000000001724. 33443366

[ref15] Hogan B. V.; Peter M. B.; Shenoy H. G.; Horgan K.; Hughes T. A. Surgery induced immunosuppression. Surgeon 2011, 9 (1), 38–43. 10.1016/j.surge.2010.07.011. 21195330

[ref16] Paruk F.; Chausse J. M. Monitoring the post surgery inflammatory host response. Journal of Emergency and Critical Care Medicine 2019, 3, 47–47. 10.21037/jeccm.2019.08.06.

[ref17] Hotchkiss R. S.; Coopersmith C. M.; McDunn J. E.; Ferguson T. A. The sepsis seesaw: tilting toward immunosuppression. Nat. Med. 2009, 15 (5), 496–497. 10.1038/nm0509-496. 19424209 PMC3786779

[ref18] Ward N. S.; Casserly B.; Ayala A. The Compensatory Anti-inflammatory Response Syndrome (CARS) in Critically Ill Patients. Clinics in Chest Medicine 2008, 29 (4), 617–625. 10.1016/j.ccm.2008.06.010. 18954697 PMC2786900

[ref19] van der Poll T.; Shankar-Hari M.; Wiersinga W. J. The immunology of sepsis. Immunity 2021, 54 (11), 2450–2464. 10.1016/j.immuni.2021.10.012. 34758337

[ref20] Kyriazopoulou E.; Hasin-Brumshtein Y.; Midic U.; Poulakou G.; Milionis H.; Metallidis S.; Astriti M.; Fragkou A.; Rapti A.; Taddei E.; et al. Transitions of blood immune endotypes and improved outcome by anakinra in COVID-19 pneumonia: an analysis of the SAVE-MORE randomized controlled trial. Critical Care 2024, 28 (1), 73 10.1186/s13054-024-04852-z. 38475786 PMC10935809

[ref21] Brakenridge S. C.; Wang Z.; Cox M.; Raymond S.; Hawkins R.; Darden D.; Ghita G.; Brumback B.; Cuschieri J.; Maier R. V.; et al. Distinct immunologic endotypes are associated with clinical trajectory after severe blunt trauma and hemorrhagic shock. J. Trauma Acute Care Surg 2021, 90 (2), 257–267. 10.1097/TA.0000000000003029. 33214489 PMC8194286

[ref22] Parente J. D.; Chase J. G.; Moeller K.; Shaw G. M. High Inter-Patient Variability in Sepsis Evolution: A Hidden Markov Model Analysis. Computer Methods and Programs in Biomedicine 2021, 201, 105956 10.1016/j.cmpb.2021.105956. 33561709

[ref23] Shimabukuro-Vornhagen A.; Gödel P.; Subklewe M.; Stemmler H. J.; Schlößer H. A.; Schlaak M.; Kochanek M.; Böll B.; von Bergwelt-Baildon M. S. Cytokine release syndrome. J. Immunother Cancer 2018, 6 (1), 56 10.1186/s40425-018-0343-9. 29907163 PMC6003181

[ref24] Singer M.; Deutschman C. S.; Seymour C. W.; Shankar-Hari M.; Annane D.; Bauer M.; Bellomo R.; Bernard G. R.; Chiche J.-D.; Coopersmith C. M.; et al. The Third International Consensus Definitions for Sepsis and Septic Shock (Sepsis-3). JAMA 2016, 315 (8), 801–810. 10.1001/jama.2016.0287. 26903338 PMC4968574

[ref25] Basile-Filho A.; Lago A. F.; Menegueti M. G.; Nicolini E. A.; Rodrigues L. A. B.; Nunes R. S.; Auxiliadora-Martins M.; Ferez M. A. The use of APACHE II, SOFA, SAPS 3, C-reactive protein/albumin ratio, and lactate to predict mortality of surgical critically ill patients: A retrospective cohort study. Medicine (Baltimore) 2019, 98 (26), e16204 10.1097/MD.0000000000016204. 31261567 PMC6617482

[ref26] Santomasso B. D.; Nastoupil L. J.; Adkins S.; Lacchetti C.; Schneider B. J.; Anadkat M.; Atkins M. B.; Brassil K. J.; Caterino J. M.; Chau I.; et al. Management of Immune-Related Adverse Events in Patients Treated With Chimeric Antigen Receptor T-Cell Therapy: ASCO Guideline. Journal of Clinical Oncology 2021, 39 (35), 3978–3992. 10.1200/JCO.21.01992. 34724386

[ref27] Evans L.; Rhodes A.; Alhazzani W.; Antonelli M.; Coopersmith C. M.; French C.; Machado F. R.; McIntyre L.; Ostermann M.; Prescott H. C.; et al. Surviving sepsis campaign: international guidelines for management of sepsis and septic shock 2021. Intensive Care Medicine 2021, 47 (11), 1181–1247. 10.1007/s00134-021-06506-y. 34599691 PMC8486643

[ref28] Millán O.; Brunet M. Cytokine-based immune monitoring. Clinical Biochemistry 2016, 49 (4), 338–346. 10.1016/j.clinbiochem.2016.01.004. 26800778

[ref29] Teachey D. T.; Lacey S. F.; Shaw P. A.; Melenhorst J. J.; Maude S. L.; Frey N.; Pequignot E.; Gonzalez V. E.; Chen F.; Finklestein J.; et al. Identification of Predictive Biomarkers for Cytokine Release Syndrome after Chimeric Antigen Receptor T-cell Therapy for Acute Lymphoblastic Leukemia. Cancer Discovery 2016, 6 (6), 664–679. 10.1158/2159-8290.CD-16-0040. 27076371 PMC5448406

[ref30] van Engelen T. S. R.; Wiersinga W. J.; Scicluna B. P.; van der Poll T. Biomarkers in Sepsis. Critical Care Clinics 2018, 34 (1), 139–152. 10.1016/j.ccc.2017.08.010. 29149935

[ref31] Somers W.; Stahl M.; Seehra J. S. 1.9 Å crystal structure of interleukin 6: implications for a novel mode of receptor dimerization and signaling. EMBO Journal 1997, 16 (5), 989–997. 10.1093/emboj/16.5.989. 9118960 PMC1169699

[ref32] Dama P.; Ledoux D.; Nys M.; Vrindts Y.; De Groote D.; Franchimont P.; Lamy M. Cytokine serum level during severe sepsis in human IL-6 as a marker of severity. Ann. Surg 1992, 215 (4), 356–362. 10.1097/00000658-199204000-00009. 1558416 PMC1242452

[ref33] Wunder C.; Eichelbrönner O.; Roewer N. Are IL-6, IL-10 and PCT plasma concentrations reliable for outcome prediction in severe sepsis? A comparison with APACHE III and SAPS II. Inflammation Res. 2004, 53 (4), 158–163. 10.1007/s00011-003-1239-3. 15060722

[ref34] Oda S.; Hirasawa H.; Shiga H.; Nakanishi K.; Matsuda K.-i.; Nakamua M. Sequential measurement of IL-6 blood levels in patients with systemic inflammatory response syndrome (SIRS)/sepsis. Cytokine 2005, 29 (4), 169–175. 10.1016/j.cyto.2004.10.010. 15652449

[ref35] Bozza F. A.; Salluh J. I.; Japiassu A. M.; Soares M.; Assis E. F.; Gomes R. N.; Bozza M. T.; Castro-Faria-Neto H. C.; Bozza P. T. Cytokine profiles as markers of disease severity in sepsis: a multiplex analysis. Crit Care 2007, 11 (2), R49 10.1186/cc5783. 17448250 PMC2206478

[ref36] Mera S.; Tatulescu D.; Cismaru C.; Bondor C.; Slavcovici A.; Zanc V.; Carstina D.; Oltean M. Multiplex cytokine profiling in patients with sepsis. APMIS 2011, 119 (2), 155–163. 10.1111/j.1600-0463.2010.02705.x. 21208283

[ref37] Gentile L. F.; Cuenca A. G.; Vanzant E. L.; Efron P. A.; McKinley B.; Moore F.; Moldawer L. L. Is there value in plasma cytokine measurements in patients with severe trauma and sepsis?. Methods 2013, 61 (1), 3–9. 10.1016/j.ymeth.2013.04.024. 23669589 PMC3683386

[ref38] Song J.; Park D. W.; Moon S.; Cho H.-J.; Park J. H.; Seok H.; Choi W. S. Diagnostic and prognostic value of interleukin-6, pentraxin 3, and procalcitonin levels among sepsis and septic shock patients: a prospective controlled study according to the Sepsis-3 definitions. BMC Infectious Diseases 2019, 19 (1), 968 10.1186/s12879-019-4618-7. 31718563 PMC6852730

[ref39] Chi Y.; Ge Y.; Wu B.; Zhang W.; Wu T.; Wen T.; Liu J.; Guo X.; Huang C.; Jiao Y.; et al. Serum Cytokine and Chemokine Profile in Relation to the Severity of Coronavirus Disease 2019 in China. J. Infect Dis 2020, 222 (5), 746–754. 10.1093/infdis/jiaa363. 32563194 PMC7337752

[ref40] Del Valle D. M.; Kim-Schulze S.; Huang H. H.; Beckmann N. D.; Nirenberg S.; Wang B.; Lavin Y.; Swartz T. H.; Madduri D.; Stock A.; et al. An inflammatory cytokine signature predicts COVID-19 severity and survival. Nat. Med. 2020, 26 (10), 1636–1643. 10.1038/s41591-020-1051-9. 32839624 PMC7869028

[ref41] Leisman D. E.; Ronner L.; Pinotti R.; Taylor M. D.; Sinha P.; Calfee C. S.; Hirayama A. V.; Mastroiani F.; Turtle C. J.; Harhay M. O.; et al. Cytokine elevation in severe and critical COVID-19: a rapid systematic review, meta-analysis, and comparison with other inflammatory syndromes. Lancet Respiratory Medicine 2020, 8 (12), 1233–1244. 10.1016/S2213-2600(20)30404-5. 33075298 PMC7567529

[ref42] Matsumoto H.; Ogura H.; Shimizu K.; Ikeda M.; Hirose T.; Matsuura H.; Kang S.; Takahashi K.; Tanaka T.; Shimazu T. The clinical importance of a cytokine network in the acute phase of sepsis. Sci. Rep 2018, 8 (1), 13995 10.1038/s41598-018-32275-8. 30228372 PMC6143513

[ref43] Kiers D.; Leijte G. P.; Gerretsen J.; Zwaag J.; Kox M.; Pickkers P. Comparison of different lots of endotoxin and evaluation of in vivo potency over time in the experimental human endotoxemia model. Innate Immunity 2019, 25 (1), 34–45. 10.1177/1753425918819754. 30782041 PMC6830888

[ref44] Jansen A.; Waalders N. J. B.; van Lier D. P. T.; Kox M.; Pickkers P. CytoSorb hemoperfusion markedly attenuates circulating cytokine concentrations during systemic inflammation in humans in vivo. Crit Care 2023, 27 (1), 117 10.1186/s13054-023-04391-z. 36945034 PMC10029173

[ref45] Taudorf S.; Krabbe K. S.; Berg R. M. G.; Pedersen B. K.; Møller K. Human Models of Low-Grade Inflammation: Bolus versus Continuous Infusion of Endotoxin. Clinical and Vaccine Immunology 2007, 14 (3), 250–255. 10.1128/CVI.00380-06. 17267590 PMC1828854

[ref46] Rella J. M.; Jilma B.; Fabry A.; Kaynar A. M.; Mayr F. B. MMP-8 genotypes influence the inflammatory response in human endotoxemia. Inflammation 2014, 37 (2), 451–456. 10.1007/s10753-013-9758-0. 24170307 PMC7101851

[ref47] van Eijk L. T.; van der Pluijm R. W.; Ramakers B. P.; Dorresteijn M. J.; van der Hoeven J. G.; Kox M.; Pickkers P. Body mass index is not associated with cytokine induction during experimental human endotoxemia. Innate Immunity 2014, 20 (1), 61–67. 10.1177/1753425913481821. 23606514

[ref48] Buttenschoen K.; Buttenschoen D. C.; Berger D.; Vasilescu C.; Schafheutle S.; Goeltenboth B.; Seidelmann M.; Beger H. G. Endotoxemia and acute-phase proteins in major abdominal surgery. American Journal of Surgery 2001, 181 (1), 36–43. 10.1016/S0002-9610(00)00534-1. 11248174

[ref49] Eck M. J.; Sprang S. R. The structure of tumor necrosis factor-alpha at 2.6 A resolution. J. Biol. Chem. 1989, 264 (29), 17595–17605. 10.1016/S0021-9258(18)71533-0. 2551905

[ref50] Gharamti A. A.; Samara O.; Monzon A.; Montalbano G.; Scherger S.; DeSanto K.; Chastain D. B.; Sillau S.; Montoya J. G.; Franco-Paredes C.; et al. Proinflammatory cytokines levels in sepsis and healthy volunteers, and tumor necrosis factor-alpha associated sepsis mortality: A systematic review and meta-analysis. Cytokine 2022, 158, 156006 10.1016/j.cyto.2022.156006. 36044827

[ref51] Clore G. M.; Appella E.; Yamada M.; Matsushima K.; Gronenborn A. M. Three-dimensional structure of interleukin 8 in solution. Biochemistry 1990, 29 (7), 1689–1696. 10.1021/bi00459a004. 2184886

[ref52] Tedesco V. E.; Mohan C. Biomarkers for Predicting Cytokine Release Syndrome following CD19-Targeted CAR T Cell Therapy. J. Immunol. 2021, 206 (7), 1561–1568. 10.4049/jimmunol.2001249. 33692146

[ref53] Hay K. A.; Hanafi L. A.; Li D.; Gust J.; Liles W. C.; Wurfel M. M.; Lopez J. A.; Chen J.; Chung D.; Harju-Baker S.; et al. Kinetics and biomarkers of severe cytokine release syndrome after CD19 chimeric antigen receptor-modified T-cell therapy. Blood 2017, 130 (21), 2295–2306. 10.1182/blood-2017-06-793141. 28924019 PMC5701525

[ref54] Yoon S. I.; Logsdon N. J.; Sheikh F.; Donnelly R. P.; Walter M. R. Conformational Changes Mediate Interleukin-10 Receptor 2 (IL-10R2) Binding to IL-10 and Assembly of the Signaling Complex*. J. Biol. Chem. 2006, 281 (46), 35088–35096. 10.1074/jbc.M606791200. 16982608

[ref55] Ishikawa S.; Teshima Y.; Otsubo H.; Shimazui T.; Nakada T. A.; Takasu O.; Matsuda K.; Sasaki J.; Nabeta M.; Moriguchi T.; et al. Risk prediction of biomarkers for early multiple organ dysfunction in critically ill patients. BMC Emerg Med. 2021, 21 (1), 132 10.1186/s12873-021-00534-z. 34749673 PMC8573766

[ref56] Tanak A. S.; Sardesai A.; Muthukumar S.; Krishnan S.; Striegel D. A.; Schully K. L.; Clark D. V.; Prasad S. Multiplexed host immune response biosensor for rapid sepsis stratification and endotyping at point-of-care. Biosensors and Bioelectronics: X 2022, 10, 100144 10.1016/j.biosx.2022.100144.

[ref57] Schneider H.-G.; Thanh Lam Q. Procalcitonin for the clinical laboratory: a review. Pathology 2007, 39 (4), 383–390. 10.1080/00313020701444564. 17676478

[ref58] Reinhart K.; Bauer M.; Riedemann N. C.; Hartog C. S. New approaches to sepsis: molecular diagnostics and biomarkers. Clin Microbiol Rev. 2012, 25 (4), 609–634. 10.1128/CMR.00016-12. 23034322 PMC3485751

[ref59] Covington E. W.; Roberts M. Z.; Dong J. Procalcitonin Monitoring as a Guide for Antimicrobial Therapy: A Review of Current Literature. Pharmacotherapy 2018, 38 (5), 569–581. 10.1002/phar.2112. 29604109

[ref60] Mazaheri T.; Ranasinghe R.; Al-Hasani W.; Luxton J.; Kearney J.; Manning A.; Dimitriadis G. K.; Mare T.; Vincent R. P. A cytokine panel and procalcitonin in COVID-19, a comparison between intensive care and non-intensive care patients. PLoS One 2022, 17 (5), e0266652 10.1371/journal.pone.0266652. 35500008 PMC9060342

[ref61] Ogasawara S.; Saito N.; Hirano R.; Minakawa S.; Kimura M.; Kayaba H. Clinical relevance of procalcitonin values in bacteremia. Journal of Infection and Chemotherapy 2020, 26 (10), 1048–1053. 10.1016/j.jiac.2020.05.023. 32595106

[ref62] Khilnani G. C.; Tiwari P.; Zirpe K. G.; Chaudhry D.; Govil D.; Dixit S.; Kulkarni A. P.; Todi S. K.; Hadda V.; Jain N.; et al. Guidelines for the Use of Procalcitonin for Rational Use of Antibiotics. Indian J. Crit Care Med. 2022, 26 (Suppl 2), S77–s94. 10.5005/jp-journals-10071-24326. 36896360 PMC9989870

[ref63] Shrive A. K.; Gheetham G. M. T.; Holden D.; Myles D. A. A.; Turnell W. G.; Volanakis J. E.; Pepys M. B.; Bloomer A. C.; Greenhough T. J. Three dimensional structure of human C-reactive protein. Nat. Struct. Biol. 1996, 3 (4), 346–354. 10.1038/nsb0496-346. 8599761

[ref64] Friedel M.; Thompson I. A. P.; Kasting G.; Polsky R.; Cunningham D.; Soh H. T.; Heikenfeld J. Opportunities and challenges in the diagnostic utility of dermal interstitial fluid. Nature Biomedical Engineering 2023, 7 (12), 1541–1555. 10.1038/s41551-022-00998-9. 36658344

[ref65] Kumari S.; Samara M.; Ampadi Ramachandran R.; Gosh S.; George H.; Wang R.; Pesavento R. P.; Mathew M. T. A Review on Saliva-Based Health Diagnostics: Biomarker Selection and Future Directions. Biomedical Materials & Devices 2024, 2 (1), 121–138. 10.1007/s44174-023-00090-z. PMC10243891 37363139

[ref66] Williamson S.; Munro C.; Pickler R.; Grap M. J.; Elswick R. K. Jr Comparison of biomarkers in blood and saliva in healthy adults. Nurs Res. Pract 2012, 2012, 246178 10.1155/2012/246178. 22619709 PMC3350846

[ref67] Andreasen A.; Krabbe K.; Krogh-Madsen R.; Taudorf S.; Pedersen B.; Moller K. Human Endotoxemia as a Model of Systemic Inflammation. Curr. Med. Chem. 2008, 15 (17), 1697–1705. 10.2174/092986708784872393. 18673219

[ref68] Calvano S. E.; Coyle S. M. Experimental Human Endotoxemia: A Model of the Systemic Inflammatory Response Syndrome?. Surgical Infections 2012, 13 (5), 293–299. 10.1089/sur.2012.155. 23072275 PMC3503465

[ref69] van Lier D.; Geven C.; Leijte G. P.; Pickkers P. Experimental human endotoxemia as a model of systemic inflammation. Biochimie 2019, 159, 99–106. 10.1016/j.biochi.2018.06.014. 29936295

[ref70] De Benedetti F.; Prencipe G.; Bracaglia C.; Marasco E.; Grom A. A. Targeting interferon-γ in hyperinflammation: opportunities and challenges. Nature Reviews Rheumatology 2021, 17 (11), 678–691. 10.1038/s41584-021-00694-z. 34611329

[ref71] Mueller A. A.; Tamura T.; Crowley C. P.; DeGrado J. R.; Haider H.; Jezmir J. L.; Keras G.; Penn E. H.; Massaro A. F.; Kim E. Y. Inflammatory Biomarker Trends Predict Respiratory Decline in COVID-19 Patients. Cell Rep. Med. 2020, 1 (8), 100144 10.1016/j.xcrm.2020.100144. 33163981 PMC7598305

[ref72] Tjendra Y.; Al Mana A. F.; Espejo A. P.; Akgun Y.; Millan N. C.; Gomez-Fernandez C.; Cray C. Predicting Disease Severity and Outcome in COVID-19 Patients: A Review of Multiple Biomarkers. Archives of Pathology & Laboratory Medicine 2020, 144 (12), 1465–1474. 10.5858/arpa.2020-0471-SA. 32818235

[ref73] Vasbinder A.; Padalia K.; Pizzo I.; Machado K.; Catalan T.; Presswalla F.; Anderson E.; Ismail A.; Hutten C.; Huang Y.; et al. SuPAR, biomarkers of inflammation, and severe outcomes in patients hospitalized for COVID-19: The International Study of Inflammation in COVID-19. Journal of Medical Virology 2024, 96 (1), e29389 10.1002/jmv.29389. 38235904 PMC10829525

[ref74] Shannon C. E. Communication Theory of Secrecy Systems*. Bell System Technical Journal 1949, 28 (4), 656–715. 10.1002/j.1538-7305.1949.tb00928.x.

[ref75] Bergkamp M. H.; Cajigas S.; van Ijzendoorn L. J.; Prins M. W. J. Real-time continuous monitoring of dynamic concentration profiles studied with biosensing by particle motion. Lab Chip 2023, 23 (20), 4600–4609. 10.1039/D3LC00410D. 37772830

[ref76] Chkroun C.; Trouwborst I.; Cherta-Murillo A.; Owen L.; Darimont C.; Rytz A. Defining a Continuous Glucose Baseline to assess the impact of nutritional interventions. Front Nutr 2023, 10, 1203899 10.3389/fnut.2023.1203899. 37588050 PMC10425768

[ref77] Lubken R. M.; Lin Y.-T.; Haenen S. R. R.; Bergkamp M. H.; Yan J.; Nommensen P. A.; Prins M. W. J. Continuous Biosensor Based on Particle Motion: How Does the Concentration Measurement Precision Depend on Time Scale?. ACS Sensors 2024, 9, 4924 10.1021/acssensors.4c01586. 39166946 PMC11443519

[ref78] Saateh A.; Ansaryan S.; Gao J.; Oliveira de Miranda L.; Zijlstra P.; Altug H. Long-term and Continuous Plasmonic Oligonucleotide Monitoring Enabled by Regeneration Approach. Angew. Chem., Int. Ed. 2024, 63 (48), e202410076 10.1002/anie.202410076. 39146470

[ref79] Ronkainen N. J.; Halsall H. B.; Heineman W. R. Electrochemical biosensors. Chem. Soc. Rev. 2010, 39 (5), 1747–1763. 10.1039/b714449k. 20419217

[ref80] Borisov S. M.; Wolfbeis O. S. Optical Biosensors. Chem. Rev. 2008, 108 (2), 423–461. 10.1021/cr068105t. 18229952

[ref81] Xiao Y.; Lubin A. A.; Heeger A. J.; Plaxco K. W. Label-free electronic detection of thrombin in blood serum by using an aptamer-based sensor. Angew. Chem., Int. Ed. Engl. 2005, 44 (34), 5456–5459. 10.1002/anie.200500989. 16044476

[ref82] Swensen J. S.; Xiao Y.; Ferguson B. S.; Lubin A. A.; Lai R. Y.; Heeger A. J.; Plaxco K. W.; Soh H. T. Continuous, Real-Time Monitoring of Cocaine in Undiluted Blood Serum via a Microfluidic, Electrochemical Aptamer-Based Sensor. J. Am. Chem. Soc. 2009, 131 (12), 4262–4266. 10.1021/ja806531z. 19271708 PMC2715559

[ref83] Li H.; Dauphin-Ducharme P.; Arroyo-Currás N.; Tran C. H.; Vieira P. A.; Li S.; Shin C.; Somerson J.; Kippin T. E.; Plaxco K. W. A Biomimetic Phosphatidylcholine-Terminated Monolayer Greatly Improves the In Vivo Performance of Electrochemical Aptamer-Based Sensors. Angew. Chem., Int. Ed. 2017, 56 (26), 7492–7495. 10.1002/anie.201700748. PMC5660315 28371090

[ref84] Li S.; Dai J.; Zhu M.; Arroyo-Currás N.; Li H.; Wang Y.; Wang Q.; Lou X.; Kippin T. E.; Wang S.; et al. Implantable Hydrogel-Protective DNA Aptamer-Based Sensor Supports Accurate, Continuous Electrochemical Analysis of Drugs at Multiple Sites in Living Rats. ACS Nano 2023, 17 (18), 18525–18538. 10.1021/acsnano.3c06520. 37703911

[ref85] Li H.; Arroyo-Currás N.; Kang D.; Ricci F.; Plaxco K. W. Dual-Reporter Drift Correction To Enhance the Performance of Electrochemical Aptamer-Based Sensors in Whole Blood. J. Am. Chem. Soc. 2016, 138 (49), 15809–15812. 10.1021/jacs.6b08671. 27960346

[ref86] Downs A. M.; Gerson J.; Hossain M. N.; Ploense K.; Pham M.; Kraatz H. B.; Kippin T.; Plaxco K. W. Nanoporous Gold for the Miniaturization of In Vivo Electrochemical Aptamer-Based Sensors. ACS Sens 2021, 6 (6), 2299–2306. 10.1021/acssensors.1c00354. 34038076 PMC12045558

[ref87] Leung K. K.; Gerson J.; Emmons N.; Heemstra J. M.; Kippin T. E.; Plaxco K. W. The Use of Xenonucleic Acids Significantly Reduces the In Vivo Drift of Electrochemical Aptamer-Based Sensors. Angew. Chem. 2024, 136 (21), e202316678 10.1002/ange.202316678. PMC11821280 38500260

[ref88] Gerson J.; Erdal M. K.; McDonough M. H.; Ploense K. L.; Dauphin-Ducharme P.; Honeywell K. M.; Leung K. K.; Arroyo-Curras N.; Gibson J. M.; Emmons N. A.; et al. High-precision monitoring of and feedback control over drug concentrations in the brains of freely moving rats. Science Advances 2023, 9 (20), eadg3254 10.1126/sciadv.adg3254. 37196087 PMC10191434

[ref89] van Smeden L.; Saris A.; Sergelen K.; de Jong A. M.; Yan J.; Prins M. W. J. Reversible Immunosensor for the Continuous Monitoring of Cortisol in Blood Plasma Sampled with Microdialysis. ACS Sensors 2022, 7 (10), 3041–3048. 10.1021/acssensors.2c01358. 36255855 PMC9623578

[ref90] Thompson I. A. P.; Saunders J.; Zheng L.; Hariri A. A.; Maganzini N.; Cartwright A. P.; Pan J.; Yee S.; Dory C.; Eisenstein M.; et al. An antibody-based molecular switch for continuous small-molecule biosensing. Sci. Adv. 2023, 9 (38), eadh4978 10.1126/sciadv.adh4978. 37738337 PMC10516488

[ref91] Arroyo-Currás N.; Dauphin-Ducharme P.; Ortega G.; Ploense K. L.; Kippin T. E.; Plaxco K. W. Subsecond-Resolved Molecular Measurements in the Living Body Using Chronoamperometrically Interrogated Aptamer-Based Sensors. ACS Sens 2018, 3 (2), 360–366. 10.1021/acssensors.7b00787. 29124939

[ref92] Parolo C.; Idili A.; Ortega G.; Csordas A.; Hsu A.; Arroyo-Currás N.; Yang Q.; Ferguson B. S.; Wang J.; Plaxco K. W. Real-Time Monitoring of a Protein Biomarker. ACS Sensors 2020, 5 (7), 1877–1881. 10.1021/acssensors.0c01085. 32619092 PMC8088336

[ref93] Chung J.; Sepunaru L.; Plaxco K. W. On the Disinfection of Electrochemical Aptamer-Based Sensors. ECS Sensors Plus 2022, 1 (1), 011604 10.1149/2754-2726/ac60b2. 36452064 PMC9703871

[ref94] Downs A. M.; Plaxco K. W. Real-Time, In Vivo Molecular Monitoring Using Electrochemical Aptamer Based Sensors: Opportunities and Challenges. ACS Sensors 2022, 7 (10), 2823–2832. 10.1021/acssensors.2c01428. 36205360 PMC9840907

[ref95] Chamorro-Garcia A.; Gerson J.; Flatebo C.; Fetter L.; Downs A. M.; Emmons N.; Ennis H. L.; Milosavić N.; Yang K.; Stojanovic M.; et al. Real-Time, Seconds-Resolved Measurements of Plasma Methotrexate In Situ in the Living Body. ACS Sensors 2023, 8 (1), 150–157. 10.1021/acssensors.2c01894. 36534756

[ref96] Verrinder E.; Leung K. K.; Erdal M. K.; Sepunaru L.; Plaxco K. W. Comparison of voltammetric methods used in the interrogation of electrochemical aptamer-based sensors. Sensors & Diagnostics 2024, 3 (1), 95–103. 10.1039/D3SD00083D. PMC13099078 42022238

[ref97] Seo J.-W.; Fu K.; Correa S.; Eisenstein M.; Appel E. A.; Soh H. T. Real-time monitoring of drug pharmacokinetics within tumor tissue in live animals. Science Advances 2022, 8 (1), eabk2901 10.1126/sciadv.abk2901. 34995112 PMC8741190

[ref98] Belmonte I.; White R. J. 3-D printed microfluidics for rapid prototyping and testing of electrochemical, aptamer-based sensor devices under flow conditions. Anal. Chim. Acta 2022, 1192, 339377 10.1016/j.aca.2021.339377. 35057946 PMC8931854

[ref99] Tsai Y.-C.; Weng W.-Y.; Yeh Y.-T.; Chien J.-C. Dual-Aptamer Drift Canceling Techniques to Improve Long-Term Stability of Real-Time Structure-Switching Aptasensors. ACS Sensors 2023, 8 (9), 3380–3388. 10.1021/acssensors.3c00509. 37671977

[ref100] Visser E. W. A.; Yan J.; van Ijzendoorn L. J.; Prins M. W. J. Continuous biomarker monitoring by particle mobility sensing with single molecule resolution. Nat. Commun. 2018, 9 (1), 2541 10.1038/s41467-018-04802-8. 29959314 PMC6026194

[ref101] Yan J.; van Smeden L.; Merkx M.; Zijlstra P.; Prins M. W. J. Continuous Small-Molecule Monitoring with a Digital Single-Particle Switch. ACS Sensors 2020, 5 (4), 1168–1176. 10.1021/acssensors.0c00220. 32189498 PMC8177406

[ref102] Lubken R. M.; de Jong A. M.; Prins M. W. J. Multiplexed Continuous Biosensing by Single-Molecule Encoded Nanoswitches. Nano Lett. 2020, 20 (4), 2296–2302. 10.1021/acs.nanolett.9b04561. 32091908 PMC7252944

[ref104] Lubken R. M.; Bergkamp M. H.; de Jong A. M.; Prins M. W. J. Sensing Methodology for the Rapid Monitoring of Biomolecules at Low Concentrations over Long Time Spans. ACS Sensors 2021, 6 (12), 4471–4481. 10.1021/acssensors.1c01991. 34854303 PMC8715529

[ref105] Buskermolen A. D.; Lin Y.-T.; van Smeden L.; van Haaften R. B.; Yan J.; Sergelen K.; de Jong A. M.; Prins M. W. J. Continuous biomarker monitoring with single molecule resolution by measuring free particle motion. Nat. Commun. 2022, 13 (1), 6052 10.1038/s41467-022-33487-3. 36229441 PMC9561105

[ref106] Lubken R. M.; de Jong A. M.; Prins M. W. J. Real-Time Monitoring of Biomolecules: Dynamic Response Limits of Affinity-Based Sensors. ACS Sensors 2022, 7 (1), 286–295. 10.1021/acssensors.1c02307. 34978190 PMC8805115

[ref107] van Smeden L.; de Jong A. M.; Prins M. W. J. Integrated sampling-and-sensing using microdialysis and biosensing by particle motion for continuous cortisol monitoring. Sensors & Diagnostics 2023, 2 (6), 1638–1648. 10.1039/D3SD00185G.

[ref108] Michielsen C. M. S.; Buskermolen A. D.; de Jong A. M.; Prins M. W. J. Sandwich Immunosensor Based on Particle Motion: How Do Reactant Concentrations and Reaction Pathways Determine the Time-Dependent Response of the Sensor?. ACS Sensors 2023, 8 (11), 4216–4225. 10.1021/acssensors.3c01549. 37955441 PMC10683507

[ref109] Buskermolen A. D.; Michielsen C. M. S.; de Jong A. M.; Prins M. W. J. Towards continuous monitoring of TNF-α at picomolar concentrations using biosensing by particle motion. Biosens. Bioelectron. 2024, 249, 115934 10.1016/j.bios.2023.115934. 38215637

[ref110] Cajigas S.; de Jong A. M.; Yan J.; Prins M. W. J. Molecular Origins of Long-Term Changes in a Competitive Continuous Biosensor with Single-Molecule Resolution. ACS Sensors 2024, 9, 3520 10.1021/acssensors.4c00107. 38967449 PMC11287755

[ref111] Park C. H.; Thompson I. A. P.; Newman S. S.; Hein L. A.; Lian X.; Fu K. X.; Pan J.; Eisenstein M.; Soh H. T. Real-Time Spatiotemporal Measurement of Extracellular Signaling Molecules Using an Aptamer Switch-Conjugated Hydrogel Matrix. Adv. Mater. 2024, 36 (4), 2306704 10.1002/adma.202306704. 37947789

[ref112] Kong D.; Thompson I. A. P.; Maganzini N.; Eisenstein M.; Soh H. T. Aptamer–Antibody Chimera Sensors for Sensitive, Rapid, and Reversible Molecular Detection in Complex Samples. ACS Sensors 2024, 9 (3), 1168–1177. 10.1021/acssensors.3c01638. 38407035

[ref113] Hariri A. A.; Cartwright A. P.; Dory C.; Gidi Y.; Yee S.; Thompson I. A. P.; Fu K. X.; Yang K.; Wu D.; Maganzini N.; et al. Modular Aptamer Switches for the Continuous Optical Detection of Small-Molecule Analytes in Complex Media. Adv. Mater. 2024, 36 (1), 2304410 10.1002/adma.202304410. 37975267

[ref114] Crivianu-Gaita V.; Thompson M. Aptamers, antibody scFv, and antibody Fab’ fragments: An overview and comparison of three of the most versatile biosensor biorecognition elements. Biosens Bioelectron 2016, 85, 32–45. 10.1016/j.bios.2016.04.091. 27155114

[ref115] IA. Ahmad M.; Amorim C. G.; Abu Qatouseh L. F.; Montenegro M. C. B. S. M. Nanobody-based immunodiagnostics: A systematic review of nanobody integration in diagnostics and deep insight into electrochemical immunoassays. Microchemical Journal 2024, 196, 109628 10.1016/j.microc.2023.109628.

[ref116] Liu J. K. The history of monoclonal antibody development - Progress, remaining challenges and future innovations. Ann. Med. Surg (Lond) 2014, 3 (4), 113–116. 10.1016/j.amsu.2014.09.001. 25568796 PMC4284445

[ref117] Kohlberger M.; Gadermaier G. SELEX: Critical factors and optimization strategies for successful aptamer selection. Biotechnology and Applied Biochemistry 2022, 69 (5), 1771–1792. 10.1002/bab.2244. 34427974 PMC9788027

[ref118] Tong H.-F.; Lin D.-Q.; Zhang Q.-L.; Wang R.-Z.; Yao S.-J. Molecular recognition of Fc-specific ligands binding onto the consensus binding site of IgG: insights from molecular simulation. Journal of Molecular Recognition 2014, 27 (8), 501–509. 10.1002/jmr.2373. 24984867

[ref119] Weinstein J.; Khersonsky O.; Fleishman S. J. Practically useful protein-design methods combining phylogenetic and atomistic calculations. Curr. Opin Struct Biol. 2020, 63, 58–64. 10.1016/j.sbi.2020.04.003. 32505941 PMC7289631

[ref120] Shui S.; Gainza P.; Scheller L.; Yang C.; Kurumida Y.; Rosset S.; Georgeon S.; Di Roberto R. B.; Castellanos-Rueda R.; Reddy S. T.; Correia B. E. A rational blueprint for the design of chemically-controlled protein switches. Nat. Commun. 2021, 12 (1), 5754 10.1038/s41467-021-25735-9. 34599176 PMC8486872

[ref121] Buglak A. A.; Samokhvalov A. V.; Zherdev A. V.; Dzantiev B. B. Methods and Applications of In Silico Aptamer Design and Modeling. Int. J. Mol. Sci. 2020, 21 (22), 8420 10.3390/ijms21228420. 33182550 PMC7698023

[ref122] Wolfe M.; Cramer A.; Webb S.; Goorskey E.; Chushak Y.; Mirau P.; Arroyo-Currás N.; Chávez J. L. Rational Approach to Optimizing Conformation-Switching Aptamers for Biosensing Applications. ACS Sensors 2024, 9 (2), 717–725. 10.1021/acssensors.3c02004. 38270529 PMC10897929

[ref123] Fercher C.; Jones M. L.; Mahler S. M.; Corrie S. R. Recombinant Antibody Engineering Enables Reversible Binding for Continuous Protein Biosensing. ACS Sensors 2021, 6 (3), 764–776. 10.1021/acssensors.0c01510. 33481587

[ref124] Goode J. A.; Rushworth J. V. H.; Millner P. A. Biosensor Regeneration: A Review of Common Techniques and Outcomes. Langmuir 2015, 31 (23), 6267–6276. 10.1021/la503533g. 25402969

[ref125] Jia Y.; Chen S.; Wang Q.; Li J. Recent progress in biosensor regeneration techniques. Nanoscale 2024, 16 (6), 2834–2846. 10.1039/D3NR05456J. 38291996

[ref126] Das J.; Gomis S.; Chen J. B.; Yousefi H.; Ahmed S.; Mahmud A.; Zhou W.; Sargent E. H.; Kelley S. O. Reagentless biomolecular analysis using a molecular pendulum. Nat. Chem. 2021, 13 (5), 428–434. 10.1038/s41557-021-00644-y. 33686229

[ref127] Périllat V. J.; Del Grosso E.; Berton C.; Ricci F.; Pezzato C. Controlling DNA nanodevices with light-switchable buffers. Chem. Commun. 2023, 59 (15), 2146–2149. 10.1039/D2CC06525H. PMC9933455 36727426

[ref128] Leung K. K.; Downs A. M.; Ortega G.; Kurnik M.; Plaxco K. W. Elucidating the Mechanisms Underlying the Signal Drift of Electrochemical Aptamer-Based Sensors in Whole Blood. ACS Sensors 2021, 6 (9), 3340–3347. 10.1021/acssensors.1c01183. 34491055 PMC12038169

[ref129] Breault-Turcot J.; Masson J. F. Microdialysis SPR: diffusion-gated sensing in blood. Chem. Sci. 2015, 6 (7), 4247–4254. 10.1039/C5SC00716J. 29218191 PMC5707466

[ref130] Watkins Z.; Karajic A.; Young T.; White R.; Heikenfeld J. Week-Long Operation of Electrochemical Aptamer Sensors: New Insights into Self-Assembled Monolayer Degradation Mechanisms and Solutions for Stability in Serum at Body Temperature. ACS Sensors 2023, 8 (3), 1119–1131. 10.1021/acssensors.2c02403. 36884003 PMC10443649

[ref131] Damodaran V. B.; Murthy N. S. Bio-inspired strategies for designing antifouling biomaterials. Biomaterials Research 2016, 20 (1), 18 10.1186/s40824-016-0064-4. 27326371 PMC4913429

